# Feasibility and outcomes of decentralized point-of-care CD19 CAR T cell for relapsed large B cell lymphoma in resource-limited settings

**DOI:** 10.1016/j.omton.2026.201278

**Published:** 2026-06-17

**Authors:** Koramit Suppipat, Supannikar Tawinwung, Ornnicha Sathitakorn, Chantiya Chanswangphuwana, Kanhatai Chiengthong, Mutita Surakijboworn, Thiti Asawapanumas, Phandee Watanaboonyongcharoen, Palada Pitakkitnukun, Manaschanok Tippawan, Supanat Kumjan, Udomsak Bunworasate, Nattiya Hirankarn, Kitsada Wudhikarn

**Affiliations:** 1 Center of Excellence in Cellular Immunotherapy, Chulalongkorn University, Bangkok 10330, Thailand; 2 Department of Research Affairs, Faculty of Medicine, Chulalongkorn University, Bangkok 10330, Thailand; 3 Department of Pharmacology and Physiology, Faculty of Pharmaceutical Sciences, Chulalongkorn University, Bangkok 10330, Thailand; 4 Division of Hematology and Center of Excellence in Translational Hematology, Department of Medicine, Faculty of Medicine, Chulalongkorn University, Bangkok 10330, Thailand; 5 Division of Pediatric Hematology and Oncology, Department of Pediatrics, Faculty of Medicine, Chulalongkorn University, Bangkok 10330, Thailand; 6 Center of Excellence in Pediatric Hematology/Oncology, Faculty of Medicine, Chulalongkorn University, Bangkok 10330, Thailand; 7 Department of Laboratory Medicine, Faculty of Medicine, Chulalongkorn University, Bangkok 10330, Thailand; 8 Transfusion Medicine Unit, King Chulalongkorn Memorial Hospital, Bangkok 10330, Thailand; 9 Transplant and Cellular Therapy Unit, King Chulalongkorn Memorial Hospital, Bangkok 10330, Thailand; 10 Center of Excellence in Immunology and Immune-Mediated Diseases, Department of Microbiology, Faculty of Medicine, Chulalongkorn University, Bangkok 10330, Thailand

**Keywords:** point-of-care, CD19 CAR T cell, large B cell lymphoma, decentralized, care access, resource-limited, feasibility, academic

## Abstract

CD19 chimeric antigen receptor (CAR) T cell therapy has transformed outcomes for patients with relapsed/refractory (R/R) large B cell lymphoma (LBCL), yet access to commercial products remains severely limited in low- and middle-income countries due to high cost, infrastructure requirements, and centralized manufacturing. Herein, we developed and implemented a decentralized point-of-care (POC) CAR T cell platform in Thailand using an automated closed system (CliniMACS Prodigy), integrating local manufacturing with clinical delivery. Between 2020 and 2025, 12 patients with R/R LBCL (median age 45.8 years) were treated, with a median vein-to-vein time of 12 days. Manufacturing was successful in 11 patients, while one received an out-of-specification dose. Final products demonstrated a balanced CD4:CD8 ratio and were enriched for memory T cell subsets. Cytokine release syndrome occurred in 5 patients (grade 3 in 1), and neurotoxicity in 1 patient (grade 1). At 3 months, the overall response rate was 58.3%, including 33.3% complete responses. One-year event-free and overall survival were 58.3% and 90%. The median total cost was USD 111,231. These findings demonstrate that decentralized POC CAR T cell therapy is feasible, effective, and more affordable, supporting scalable implementation in resource-limited settings.

## Graphical abstract

**Figure:**
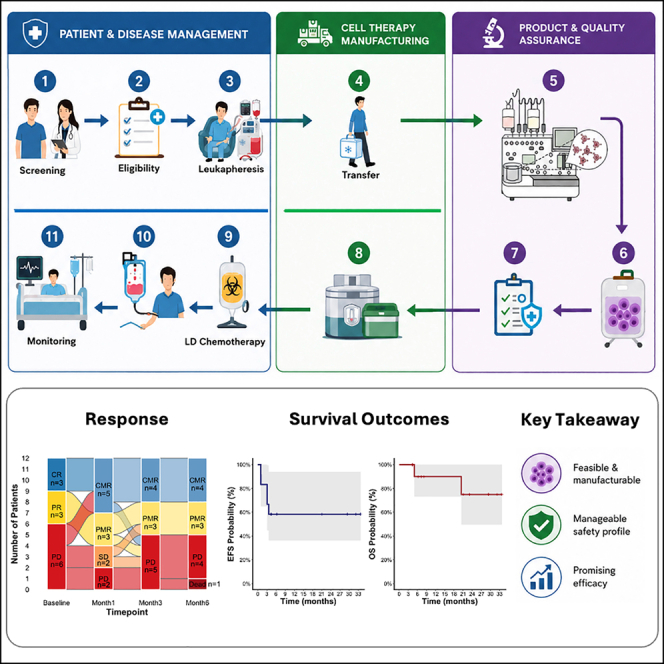

## Teaser

This study demonstrates that decentralized point-of-care CD19 CAR T cell therapy can be safely and effectively delivered in a resource-limited setting, with outcomes comparable to commercial CAR T cell products at substantially lower cost. The findings provide an important model for expanding access to advanced cellular therapies in low- and middle-income countries.

## Introduction

Approximately 35%–40% of patients with large B cell lymphoma (LBCL) experience relapse or fail to respond to standard immunochemotherapy with an anti-CD20 monoclonal antibody combined with anthracycline-based multi-agent chemotherapy.^1^^,^^2^ The prognosis for patients with relapsed or refractory (R/R) disease is particularly poor, especially among those with ultra-high-risk features, including early relapse (<12 months after first-line therapy), primary refractory disease, or nonresponse to salvage therapy.^2^^,^^3^^,^^4^ Until recently, high-dose chemotherapy followed by autologous stem cell transplantation (ASCT) had remained the standard of care for these patients, but outcomes were generally disappointing.^5^^,^^6^^,^^7^^,^^8^ Chimeric antigen receptor (CAR) T cell therapy has now emerged as a transformative treatment option, offering high response rates and the potential for durable remission in patients who previously had limited therapeutic options.^9^^,^^10^ Since 2017, three commercial CD19 CAR T cell products have been approved as standard therapy for patients with R/R LBCL.^11^^,^^12^^,^^13^ Despite their remarkable efficacy, access in resource-limited settings is nearly impossible due to prohibitive costs and the lack of a government-led implementation plan, including reimbursement and infrastructure, with regulatory policies remaining underdeveloped.^14^^,^^15^^,^^16^^,^^17^ To overcome these barriers, several point-of-care (POC) CAR T cell platforms such as automated Prodigy CliniMACS systems have been developed and implemented in several resource-constrained countries, enabling timely and more affordable delivery of this cutting-edge therapy.^18^^,^^19^ In this study, we report the feasibility, implementation, and early clinical outcomes of decentralized POC CD19 CAR T cell manufacturing and delivery, as well as the streamlined patient care process, for patients with R/R LBCL at a university medical center in Thailand.

## Results

### Patient characteristics

A total of 12 patients with R/R LBCL received CD19 CAR T cell therapy between January 2020 and December 2025. The median age at the time of CAR T cell infusion was 45.8 years (interquartile range [IQR], 21.8–63.4). All patients were treated for R/R LBCL, including de novo diffuse large B cell lymphoma (DLBCL), 2 cases of transformed DLBCL, and 1 case of primary mediastinal B cell lymphoma (PMBCL). The median number of prior lines of therapy was 3 lines (range, 2–5) with no patients undergoing ASCT prior to CAR T cell. The median interval of 61 days (IQR, 42–97) from the most recent line of treatment to CAR T cell infusion. The median time from CAR T cell consultation to CAR T cell infusion was 76 days (IQR, 46–138). Table 1 provides overall baseline demographic and clinical characteristics of patients enrolled in this cohort. The detailed patient-level baseline demographic and clinical characteristic for each of the 12 individual patients is summarized in Table 2.

**Table 1:** Demographic and baseline characteristics of patients treated with CD19 CAR T cell

Characteristics	***N* = 12**
Median age at CAR T cell infusion (year, range)	45.8 (15.3–67.8)
Male sex	6 (50%)
Diagnosis	
De novo diffuse large B cell lymphoma	9 (75%)
Primary mediastinal B cell lymphoma	1 (8.33%)
Transformed follicular lymphoma	2 (16.67%)
Disease characteristics at the time of diagnosis	
Advanced stage (stage 3 or 4)	10 (83.33%)
Elevated lactate dehydrogenase	10 (83.33%)
Two or more extra-nodal involvement	6 (50%)
Bulky disease	9 (75%)
B symptoms	5 (41.67%)
International prognostic index at diagnosis	
0–1	3 (25%)
2	2 (16.67%)
3	5 (41.67%)
4–5	2 (16.67%)
Cell of origin	
Germinal center B cell	6 (50%)
Non-germinal center B cell	5 (41.67%)
Unknown	1 (8.33%)
Disease response after first line treatment	
Primary refractory	8 (66.67%)
Early relapse (<12 months)	2 (16.67%)
Late relapse (≥12 months)	2 (16.67%)
Line of prior therapies	
1–2 lines	4 (33.33%)
3 lines	6 (50%)
≥ 4 lines	2 (16.67%)
ECOG Performance status at CAR T cell infusion	
0–1	11 (91.67%)
2	1 (8.33%)
Median time from diagnosis to CAR T cell infusion (months, range)	15.15 (9.13–32.67)
Disease characteristics before CAR T cell consultation	
Advanced stage (stage 3 or 4)	7 (58.33%)
Elevated lactate dehydrogenase	10 (83.33%)
Two or more extra-nodal involvement sites	6 (50%)
Bulky disease	4 (33.33%)

CAR, chimeric antigen receptor; ECOG, Easter Cooperative Oncology Group.

**Table 2:** Comprehensive patient-level data including baseline characteristics, treatment exposure, and CAR T cell therapy details in the 12-patient cohort

Patient ID	Age at CAR T (years)	Sex	Diagnosis	Relapse/refractory	# Of prior treatment	Prior HCT	LDH at CAR T	Disease status at CAR T	Holding therapy	Vein to vein time (days)	LD chemotherapy	Cell dose (×10^6^ cell)	Cell dose/kg (×10^6^/kg)	Duration of admission (days)
1	54	m	DLBCL	refractory	3	no	normal	PR	yes	10	flu 30 mg/m^2^ Cy 500 mg/m^2^ (D-5 to D-3)	55	1.00	27
2	65	f	DLBCL	refractory	5	no	high	PR	yes	8	flu 30 mg/m^2^ Cy 500 mg/m^2^ (D-5 to D-3)	94	1.92	28
3	40	m	DLBCL	refractory	2	no	high	PD	no	12	flu 30 mg/m^2^ Cy 500 mg/m^2^ (D-5 to D-3)	200	1.32	29
4	19	m	DLBCL	refractory	3	no	normal	PD	yes	9	flu 30 mg/m^2^ Cy 500 mg/m^2^ (D-5 to D-3)	120	1.94	34
5	15	f	DLBCL	refractory	2	no	high	PD	no	12	flu 30 mg/m^2^ Cy 500 mg/m^2^ (D-5 to D-3)	140	1.95	29
6	66	m	DLBCL (transform)	relapse	3	no	high	PD	no	12	flu 30 mg/m^2^ Cy 500 mg/m^2^ (D-5 to D-3)	52	0.66	27
7	21	f	DLBCL	relapse	2	no	normal	CR	no	11	flu 30 mg/m^2^ Cy 500 mg/m^2^ (D-5 to D-3)	104	2.08	21
8	23	m	DLBCL	refractory	3	no	high	PD	yes	11	flu 30 mg/m^2^ Cy 500 mg/m^2^ (D-5 to D-3)	100	2.15	29
9	57	m	DLBCL (transform)	refractory	2	no	normal	PR	no	13	flu 30 mg/m^2^ Cy 500 mg/m^2^ (D-5 to D-3)	116	1.96	17
10	51	f	DLBCL	relapse	3	no	high	CR	no	13	flu 30 mg/m^2^ Cy 500 mg/m^2^ (D-5 to D-3)	161	2.01	22
11	35	f	PMBCL	refractory	3	no	high	PD	yes	12	flu 30 mg/m^2^ Cy 500 mg/m^2^ (D-5 to D-3)	138	2.03	18
12	68	f	DLBCL	relapse	4	no	high	CR	yes	12	flu 30 mg/m^2^ Cy 500 mg/m^2^ (D-5 to D-3)	146	2.13	23

M, male; F, female; DLBCL, diffuse large B cell lymphoma; PMBCL, primary mediastinal B cell lymphoma; CR, complete remission; PR, partial remission; PD, progressive disease; HCT, hematopoietic cell transplantation; CAR, chimeric antigen receptor.

### Cell composition and characteristics during CAR T cell manufacturing process from leukapheresis product, enriched T cell to final CAR T cell product

**Figure 1:**
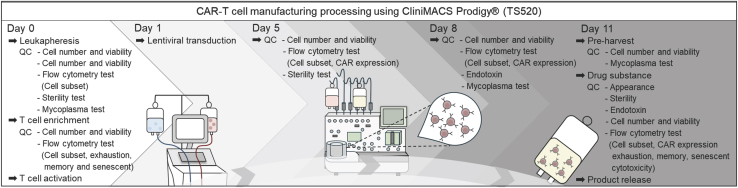
CAR T cell manufacturing workflow—Stepwise CAR T cell manufacturing process

**Figure 2:**
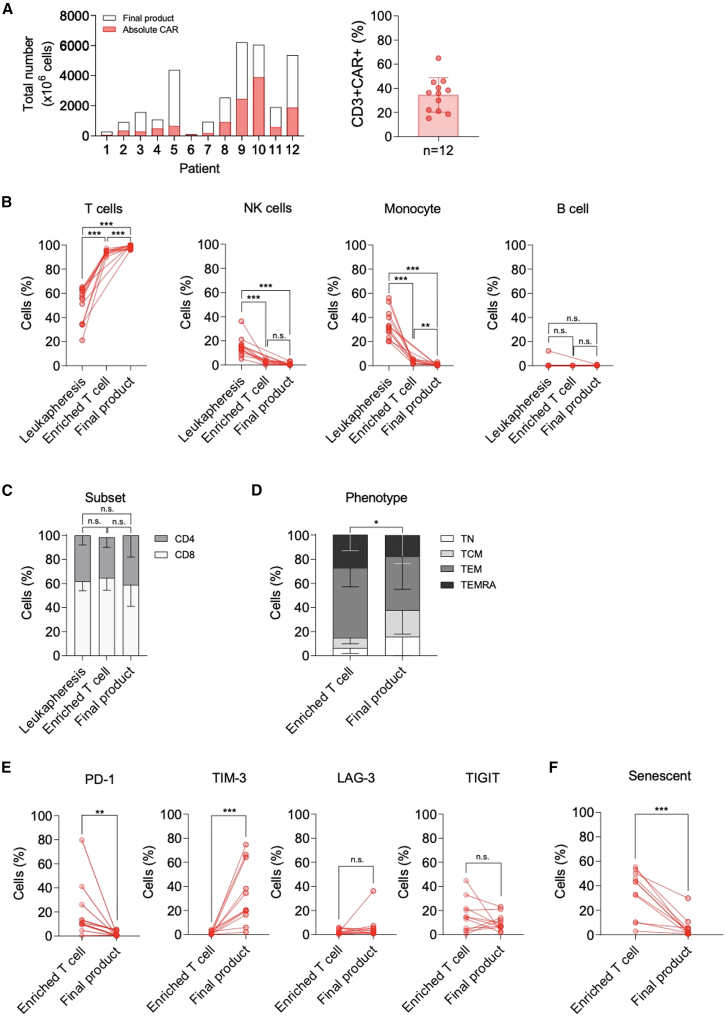
Longitudinal changes in lymphocyte composition, phenotypic characteristics, and exhaustion profiles during CAR T cell manufacturing (A) Proportions of lymphocytes and CAR^+^ cells in the final product. (B) Percentages of leukocyte subsets at different manufacturing steps. n.s., not significant (*p* > 0.05); ∗∗, *p* < 0.01; ∗∗∗, *p* < 0.001. (C) Distribution of CD4^+^ and CD8^+^ T cells across manufacturing stages. n.s., not significant (*p* > 0.05). (D) Distribution of T cell phenotypic subsets at different manufacturing steps. ∗, *p* < 0.05. (E and F) Expression of exhaustion markers and senescence-associated features across manufacturing stages. n.s., not significant (*p* > 0.05); ∗∗, *p* < 0.01; ∗∗∗, *p* < 0.001.

Clinical-grade autologous CD19 CAR T cells were successfully manufactured for all 12 enrolled patients using the CliniMACS Prodigy automated platform (Figure 1). Figure 2 demonstrates the detailed composition, phenotypic characteristics, and exhaustion profile of lymphocytes at each step of CAR T cell manufacturing. The distribution of T cell subsets was evaluated at the leukapheresis, enriched T cell, and final CAR T cell product stages. In the leukapheresis product, the median proportion of CD8^+^ T cells was 62% (range, 43–72) and CD4^+^ T cells was 38% (range, 28–57), corresponding to a median CD4:CD8 ratio of 0.61 (range, 0.39–1.33). Following enrichment, the enriched T cell fraction demonstrated a median CD8^+^ proportion of 60.5% (range, 49.5–79) and CD4^+^ proportion of 37.7% (range, 21–44), with a median CD4:CD8 ratio of 0.54 (range, 0.27–0.88). The median proportion of central memory T cells (TCMs) was 8.0% (range, 2–19), naive T cells (TNs) 5.0% (range, 1–13), effector memory T cells (TEMs) 58.0% (range, 31–86), and T effector memory cells re-expressing CD45RA (TEMRA) 28.0% (range, 9–53).

The distribution of T cell differentiation subsets was analyzed in the enriched T cell fraction and the final CAR T cell product. In the final CAR T cell product, the mean CD3^+^ T cell purity in the final product was 96.99% ± 5.35%, with a mean cell viability of 95.59% ± 2.24%. The total cell yield in the final product ranged from 126 × 10^6^ to 6,210 × 10^6^ CD3^+^ T cells, with a median yield of 1,740 × 10^6^ cells. This corresponded to a fold expansion ranging from 1.26-fold to 62.1-fold, with a median expansion of 17.4-fold during the culture period. The median CD8^+^ T cell proportion was 63.5% (range, 30–83.29) and CD4^+^ T cell proportion was 36.5% (range, 17–70), resulting in a median CD4:CD8 ratio of 0.56 (range, 0.20–2.33). In the final CAR T cell product, the median proportion of TCM was 19.0% (range, 0–67), TN 9.0% (range, 1–50), TEM 51.0% (range, 11–86), and TEMRA 7.5% (range, 0–70).

The median CAR transduction efficiency in the final product was 34.46% ± 14.16%. The absolute number of CAR-positive T cells ranged from 55 × 10^6^ to 3,908 × 10^6^ cells, with a median of 418.2 × 10^6^ cells. Functional potency testing showed a mean cytotoxic activity of 84.14% ± 26.85% against CD19-positive target cells. Vector copy number analysis demonstrated a mean of 1.67 ± 0.40 copies per cell, within predefined safety thresholds. One patient (patient #6) received a suboptimal CAR T cell dose due to limited expansion (52 × 10^6^ cells, or 0.66 × 10^6^ cells/kg). Detailed product characteristics for each patient are provided in Table S1.

### Efficacy of CAR T cell

**Figure 3:**
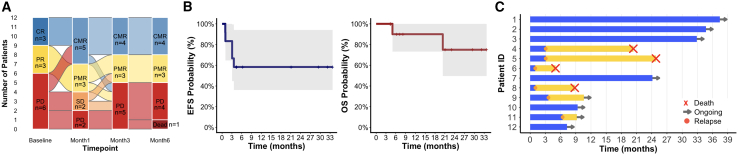
Clinical outcomes following CD19 CAR T cell therapy (A) Sankey diagram illustrating disease status transitions at each time point after therapy. (B) Event-free survival and overall survival. (C) Swimmer plot of individual patient outcomes.

The overall response rate (ORR) at 3 months was 58.3%, including a complete remission (CR) rate of 33.3% and a partial remission (PR) rate of 25% (Table 3). The best ORR achieved at any time point was 75%, comprising 50% CR and 25% PR. Three patients were in CR prior to CAR T cell infusion and remained in complete metabolic response (CMR) at 1 and 3 months post-CAR T cell therapy, corresponding to a 100% continued CR rate. Figure 3A depicts response after CAR T cell therapy after CAR T cell therapy at different time points. At a median follow-up of 14.52 months, relapse occurred in 5 patients, and 4 patients died, with disease progression as the cause of death in all cases. The 1 year event-free survival (EFS) and overall survival (OS) were 58.3% (95% confidence intervals [CI], 36.2–94.1) and 90.0% (95% CI, 73.2–100), respectively (Figure 3B). Figure 3C presents a swimmer plot depicting patient-level responses and clinical outcomes following CD19 CAR T cell therapy.

**Table 3:** Patient-level response, safety profile, and toxicities among 12 patients treated with prodigy CliniMACS CD19 CAR T cell

Patient	Response at 3 months	CRS (y/n)	CRS onset (days)	ICANS (y/n)	ICANS onset (days)	Tocilizumab (y/n)	Corticosteroid (y/n)	Late N-ICAHT (y/n)	Late T-ICAHT (y/n)	Infection (y/)
1	PMR	no	NA	grade 1	6	no	no	no	no	COVID-19 (grade 1)
2	PMR	grade 2	1	no	NA	yes	no	no	no	no
3	CMR	grade 3	1	no	NA	yes	no	no	no	no
4	PD	no	NA	no	NA	no	no	grade 1	no	no
5	PD	no	NA	no	NA	no	no	no	no	no
6	PD	no	NA	no	NA	no	no	grade 2	grade 2	no
7	CMR	no	NA	no	NA	no	no	no	no	no
8	PD	grade 1	1	no	NA	no	no	no	no	CMV viremia (grade 2)
9	PD	no	NA	no	NA	no	no	no	no	no
10	CMR	grade 1	1	no	NA	yes	no	no	grade 1	no
11	PMR	no	NA	no	NA	no	no	no	no	no
12	CMR	grade 1	5	no	NA	no	no	no	grade 1	no

CRS, cytokine release syndrome; ICANS, immune effector cell-associated neurotoxicity syndrome; ICAHT, immune effector cell-associated hematotoxicity; N, neutropenia; T, thrombocytopenic; CMV, cytomegalovirus; Y, yes; N, no; NA, not applicable; PMR, partial metabolic response; CMR, complete metabolic response; PD, progressive disease.

### Safety

Cytokine release syndrome (CRS) occurred in 5 patients (41.7%), with a median onset of 1 day after CAR T cell infusion (range, 1–5 days). One patient (20%) developed grade 3 CRS. Tocilizumab was administered in 2 patients (40%), and no patients required systemic corticosteroids. The median duration of CRS was 1 day (range, 1–5 days). Immune effector cell-associated neurotoxicity syndrome (ICANS) occurred in 1 patient (8.3%) and was limited to grade 1; no cases of severe ICANS were observed. No patients required intensive care unit (ICU) monitoring or transfer. The median length of hospitalization was 27 days (range, 17–34 days). Grade ≥3 cytopenias before day 30 were common, including neutropenia (absolute neutrophil count <1,000/μL) in 9 patients (75%) and thrombocytopenia (<50,000/μL) in 3 patients (25%). All patients with neutropenia had an absolute neutrophil count <500/μL, and 5 (55.6%) had an absolute neutrophil count <100/μL According to the immune effector cell-associated hematotoxicity (ICAHT) criteria defined by the European Hematology Association (EHA)/European Society for Blood and Marrow Transplantation (EBMT), early neutropenic ICAHT occurred in 8 patients (67%) and late neutropenic ICAHT in 7 patients (58%), with no grade ≥3 events observed. Early and late thrombocytopenic ICAHT occurred in 2 and 3 patients, respectively, without grade ≥3 toxicity. Infectious complications were observed in 2 patients (16.7%). Infectious complications occurred in 2 patients (16.7%). One patient developed cytomegalovirus reactivation without end-organ disease requiring preemptive ganciclovir therapy on day +8 after CAR T cell infusion, and another patient developed severe acute respiratory syndrome coronavirus 2 (SARS-CoV-2) infection requiring remdesivir treatment. No grade ≥3 infections were observed. Individual patient-level safety data are summarized in Table 3. Overall adverse events are summarized in Table S2.

### CAR T cell, immune monitoring, and inflammatory cytokines

**Figure 4:**
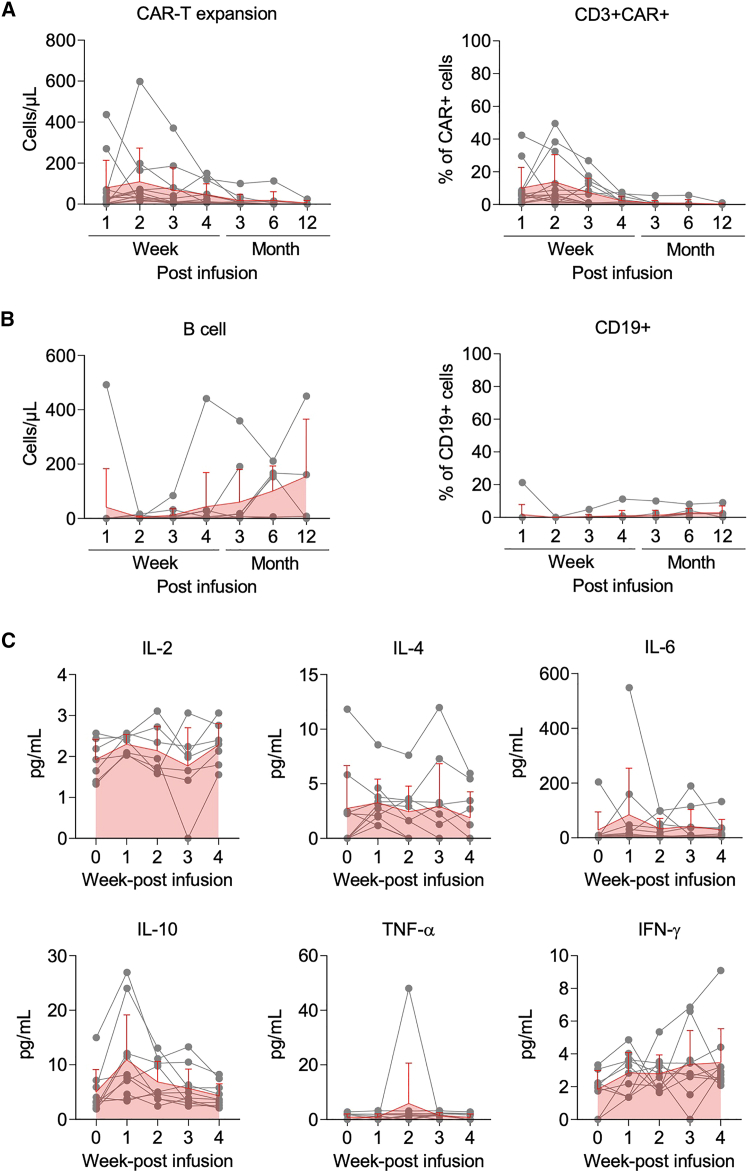
Longitudinal immune reconstitution and cytokine dynamics following CD19 CAR T cell therapy (A) CAR T cell levels measured by flow cytometry at serial time points post-infusion. (B) B cell counts measured by flow cytometry at serial time points post-infusion. (C) Longitudinal trends in cytokine levels across post-infusion time points.

Peripheral blood monitoring demonstrated early CAR T cell expansion followed by a gradual decline over time. Circulating CAR T cells were detectable in all evaluable patients by day 7 (median, 28.5 cells/μL; range, 1–437), peaking at day 14 (median, 55 cells/μL; range, 15–598), then decreasing by days 21 and 28 to medians of 21 cells/μL (range, 3–371) and 18 cells/μL (range, 5–150), respectively. Among patients with available monitoring, low-level persistence at month 3 was observed in 9 of 10 patients (median, 8 cells/μL; range, 1–100), while CAR T cells were undetectable in one patient. Persistence was observed in 4 of 7 patients at month 6 and in 1 of 4 patients at month 12 (patient #3, 24 cells/μL). Figure 4A demonstrates the CAR T cell level at each time point.

At baseline, the median circulating B cell count was 16 cells/μL (range, 0–22). Following CAR T cell infusion, rapid B cell depletion was observed, with median B cell counts decreasing to 0 cells/μL by day 7 (range, 0–492); 11 patients developed B cell aplasia (≤1 cell/μL). Sustained depletion persisted through days 14 and 21, with median counts of 0 cells/μL (range, 0–16) and 2 cells/μL (range, 0–84), respectively. By day 28, early B cell recovery (≥10 CD19-positive B cells/μL) was observed in 3 patients, with a median count of 3 cells/μL (range, 0–441). Longitudinal changes in peripheral B cell counts are shown in Figure 4B. The median immunoglobulin G (IgG) level was 680.7 (range, 500.1–2,006.6) mg/dL at baseline, 603.6 (451.4–1,148.0) mg/dL at 1 month, 536.0 (390.7–679.0) mg/dL at 3 months, 460.0 (360.9–519.5) mg/dL at 6 months, and 427.7 (358.1–700.6) mg/dL at 1 year following CAR T cell therapy (Figure S1). All patients received intravenous (i.v.) immunoglobulin replacement when IgG levels fell below 400 mg/dL in accordance with institutional protocol.

The median IL-6 level at baseline was 5.1 pg/mL (range, 2.0–204.1). By day 7, IL-6 levels increased to a median of 24.6 pg/mL (range, 3.6–548.9). At day 14, the median IL-6 level was 27.1 pg/mL (range, 3.0–99.4). By day 21, the median IL-6 level decreased to 22.8 pg/mL (range, 2.4–189.6), and by day 28, it was 23.4 pg/mL (range, 3.6–132.6). The median IL-2 levels at baseline, day 7, day 14, day 21, and day 28 were 1.93 (range, 0–2.57), 2.24 (range, 0–2.57), 1.84 (range, 0–3.11), 1.82 (range, 0–3.06), and 2.09 pg/mL (range, 0–3.06), respectively. Longitudinal trends of all measured cytokines are shown in Figure 4C.

### Expense and resource utilization of CAR T cell

**Figure 5:**
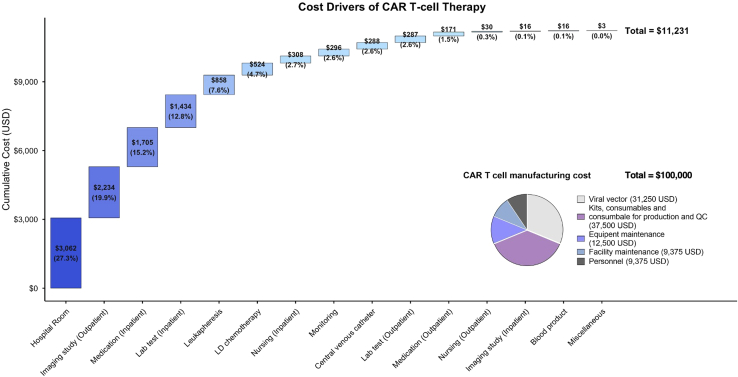
Distribution of CAR T cell-related expenses, illustrating the relative contribution of key cost drivers

Total CAR T cell-related expenses and healthcare utilization were evaluated from leukapheresis through day 30 post-CAR T cell infusion from the hospital/provider perspective. Resource utilization included leukapheresis, hospital admission, ICU utilization, blood product support, and procedure-related services. The median leukapheresis cost was 858 USD (range, 793–1,537), while the median CAR T cell manufacturing cost was 100,000 USD. The median hospital stay was 27 days (range, 17–34), and no patients required ICU transfer. Median inpatient expense through discharge was 7,609 USD (range, 4,719–13,247), with cost breakdowns shown in Figure 5. After discharge, patients were followed in the cellular therapy clinic, with outpatient costs through day +90 post-infusion or disease progression totaling a median of 2,764 USD (range, 284–4,216). Figure 5 summarizes the overall CAR T cell-related expenses.

## Discussion

In this study, we report the feasibility and early clinical outcomes of a large-scale decentralized POC CD19 CAR T cell manufacturing platform for patients with R/R LBCL in a resource-limited setting. Although several CD19 CAR T cell therapies have received US FDA and European Medicines Agency (EMA) approval since 2017 for relapsed or refractory B cell malignancies, global access remains limited, particularly in low- and middle-income countries (LMICs) and parts of Asia. This limited accessibility reflects major barriers, including high treatment costs, specialized infrastructure requirements, limited reimbursement, complex centralized manufacturing logistics, and evolving regulatory frameworks.^20^ Decentralized POC CAR T cell manufacturing may represent an important strategy to improve global access and reduce disparities in advanced cellular therapies, as demonstrated in prior studies from multiple regions worldwide (Table S4).^19^^,^^21^^,^^22^^,^^23^^,^^24^^,^^25^^,^^26^^,^^27^^,^^28^^,^^29^^,^^30^^,^^31^^,^^32^ In our cohort, 12 patients with R/R DLBCL received Miltenyi POC CD19 CAR T cell therapy, demonstrating efficacy comparable to FDA-approved products and prior POC reports, with a 3-months best ORR of 75%, 1-year EFS of 58.3%, and 1-year OS of 90%. Outcomes were also similar to those reported by Luanpitpong et al. in a prior Thai POC CD19 CAR T cell study involving nine patients with hematologic malignancies.^27^ However, that study primarily focused on feasibility and manufacturing without detailed survival or toxicity data. In contrast, our study provides a broader evaluation of clinical efficacy, survival, and safety, further supporting decentralized POC CAR T cell strategies in LMIC settings. Another study from Nguyen et al. demonstrated the feasibility of POC CD19 CAR T cell therapy in 8 patients with R/R acute lymphoblastic leukemia (ALL) and 8 patients with R/R B-non Hodgkin Lymphoma (NHL).^21^ The reported ORR in R/R B-NHL was 87.5% at 3 months, with an excellent 1 year progression free survival of 87.5%. However, unlike the study by Nguyen, our analysis focused exclusively on patients with R/R LBCL. Collectively, these findings suggest that POC CD19 CAR T cell therapy may achieve efficacy comparable to commercially available FDA-approved CAR T cell therapies,^11^^,^^12^^,^^13^^,^^33^^,^^34^^,^^35^ although cross-study comparisons should be interpreted cautiously.

Regarding healthcare utilization, Palani et al. from India reported a median healthcare cost of US $12,724 and manufacturing cost of US $35,107 among 10 patients with R/R B cell malignancies, whereas CAR T cell clinical care costs in Vietnam were higher at approximately US $40,000.^25^^,^^28^ In this cohort, enrolled patients received treatment through a pilot free-of-charge compassionate-access program established to facilitate the development and implementation of a streamlined commercial CD19 CAR T cell service. We observed substantially lower median clinical care expenses, supporting the affordability of POC CAR T cell therapy. These findings highlight the major cost advantage of POC CD19 CAR T cell manufacturing compared with commercially available products, which remain largely inaccessible in many Asian countries, including Thailand. Including clinical care costs, real-world data suggest that the total expenditure for patients with R/R LBCL receiving commercial CD19 CAR T cell therapy may approach US $700,000, representing a major barrier to access in LMIC settings.^36^^,^^37^ Collectively, these observations highlight the potential of decentralized POC CAR T cell platforms to improve affordability and expand access to cellular therapies in LMICs, where financial and infrastructure barriers limit access to commercial products. Together with emerging data from other Southeast Asian programs, these findings support decentralized POC CAR T cell manufacturing as a feasible strategy to expand access and reduce disparities in resource-limited settings. Our study has several unique strengths compared with previous reports. We provide comprehensive data on POC CD19 CAR T cell therapy in a homogeneous cohort of patients with R/R LBCL, along with detailed insights across the entire treatment continuum from manufacturing processes to health care utilization. These integrated data offer practical insights that may support the development of decentralized CAR T cell programs in LMIC settings. However, several limitations should be acknowledged. The sample size was small, reflecting the early experience of a POC CAR T cell program, and the single-center design may limit generalizability. In addition, the relatively short follow-up limits assessment of long-term durability and late toxicities. Finally, although we provided detailed analyses of healthcare utilization and costs, these findings may vary across healthcare systems and reimbursement settings. Larger multicenter studies with longer follow-up are needed to validate the clinical outcomes and economic sustainability of POC CAR T cell platforms in LMICs.

In conclusion, our study demonstrates the feasibility, safety, and promising efficacy of decentralized POC CD19 CAR T cell therapy for patients with R/R LBCL in a resource-limited setting. These findings support decentralized CAR T cell platforms as a strategy to reduce costs and expand access to advanced cellular therapies where commercial products remain limited.

## Materials and methods

### Patients eligibility, selection, and screening

We conducted a single-center retrospective cohort study of CD19 CAR T cell therapy in patients with R/R LBCL. All patients with relapsed/refractory large B cell lymphoma (R/R LBCL) who received CD19-directed CAR T cell therapy through the pilot free-of-charge compassionate access program at King Chulalongkorn Memorial Hospital between January 2020 and December 2025 were included in the analysis. All patients provided written informed consent for treatment in accordance with the institutional standard consent process. The study protocol was approved by the institutional review board (IRB no. 0869/68) and was conducted in accordance with the Declaration of Helsinki.

### CAR T cell logistics

Potential patients were initially evaluated by transplant and cellular therapy physicians (K.W., C.C., T.A., and M.S.) to determine eligibility for CD19 CAR T cell therapy. Once deemed eligible, patients were assigned to a dedicated CAR T cell coordinator, who oversaw the screening process, managed logistics related to CAR T cell manufacturing and coordinated any necessary holding therapy prior to infusion between CAR T cell physicians and primary hematologists. The screening process included a comprehensive assessment of disease status, organ function, comorbidities, and prior treatment history to ensure patient safety and optimize outcomes. After completion of screening and, if required, holding therapy, patients underwent leukapheresis for lymphocyte collection, which served as the starting material for CAR T cell production. Once leukapheresis was completed, patients were admitted to the transplant and cellular therapy unit, where they remained under close monitoring for lymphodepletion chemotherapy, CAR T cell infusion, and early post-infusion toxicities. All expense related to CAR T cell manufacturing and clinical care were supported by developmental grant from the Thai Office of the National Economic and Social Development Council, which aims to improve access to innovative therapies for Thai patients and philanthropic support. Figure S2 illustrates the workflow and logistics of CAR T cell delivery at our institution.

### Leukapheresis

Leukapheresis was performed using the Spectra Optia Apheresis System (Terumo BCT, Lakewood, CO, USA) with the continuous mononuclear cell (MNC) collection protocol according to institutional standard operating procedures. Peripheral venous access was used whenever feasible; otherwise, a temporary central venous catheter was placed. Anticoagulation was achieved using acid-citrate-dextrose solution A (ACD-A) at a ratio of 1:12 to whole blood. The inlet flow rate was maintained at 40–70 mL/min, depending on venous access and patient tolerance. A total of 5.18 L (median, range 2.88–7.87 L) of whole blood was processed during each procedure to achieve the final product volume range 70–200 mL within 4 h processing time. The leukapheresis yielded a median volume of 95 mL (range, 90–190 mL), a median MNC count of 5.15 × 10^9^ cells (range, 1.74–9.59 × 10^9^) and a median CD3^+^ T cell yield of 2.51 × 10^9^ cells (range, 0.89–4.49 × 10^9^). The collection interface and flow parameters were adjusted during the procedure to optimize MNC collection while minimizing red blood cell contamination. Patients were continuously monitored for vital signs and citrate-related symptoms throughout the procedure, and calcium supplementation was administered when clinically indicated. Leukapheresis products were processed immediately after collection without cryopreservation. Additional technical details of the leukapheresis procedure are provided in the supplemental information.

### CAR construct and manufacturing process

#### Reagents

All reagents used for CAR T cell manufacturing were clinical grade and compliant with good manufacturing practice (GMP) standards. These included CliniMACS PBS/EDTA buffer, CliniMACS CD4 MicroBeads, CliniMACS CD8 MicroBeads, T cell TransAct, and TexMACS medium supplemented with recombinant human interleukin-7 (IL-7) and interleukin-15 (IL-15) at a final concentration of 12.5 ng/mL each. Unless otherwise specified, all reagents were obtained from Miltenyi Biotec (Bergisch Gladbach, Germany). Table S3 provides a detailed list of the proteins and antibodies utilized in the CAR T cell manufacturing process in this study.

### Lentiviral vector

The clinical-grade CD19 CAR lentiviral vector used for CAR T cell manufacturing was obtained from Miltenyi Biotec for use in this pilot compassionate-access program. The CD19-directed CAR construct was delivered using a GMP-grade, replication-incompetent lentiviral vector encoding a second-generation anti-CD19 CAR. The CAR construct comprised an FMC63-derived single-chain variable fragment (scFv) targeting domain, a CD8α hinge region, a TNFRSF7 (CD27) transmembrane domain, a 4-1BB (CD137) co-stimulatory domain, and a CD3ζ intracellular signaling domain. The lentiviral vector and CAR construct were previously validated in preclinical and clinical studies demonstrating safety and functional activity.^19^^,^^38^ Figure S3 depicts the structure of the CAR lentiviral vector construct.

### CAR T cell manufacturing process

Autologous CD19 CAR T cells were manufactured using the fully automated, closed, and GMP-compliant CliniMACS Prodigy platform (Miltenyi Biotec, Bergisch Gladbach, Germany) following the T cell Transduction protocol with the TS520 tubing set, as previously described and briefly summarized here.^24^^,^^31^^,^^38^ Following leukapheresis, CD4^+^ and CD8^+^ T cells were enriched by immunomagnetic selection and subsequently activated using T cell TransAct. Activated T cells were transduced with a CD19-directed lentiviral vector and expanded in TexMACS medium supplemented with recombinant human IL-7 and IL-15 at a final concentration of 12.5 ng/mL each. The manufacturing process was completed over 8–12 days, depending on cell expansion kinetics and achievement of the predefined target cell dose. At harvest, cells were washed and formulated in 2.5% human serum albumin in normal saline for infusion. Manufacturing was performed at the Cell and Gene Therapy Manufacturing Center, King Chulalongkorn Memorial Hospital, Thai Red Cross Society, a manufacturing facility licensed by the Thai Food and Drug Administration (FDA) (manufacturing license no. 13/2567). All procedures were conducted in an ISO (International Organization for Standardizations) Class 7 cleanroom under validated cleanroom conditions and institutional quality management systems. Figure 1 illustrates the stepwise manufacturing workflow performed on the CliniMACS Prodigy platform. The flow cytometry gating strategies used to define T cell subsets, T cell phenotypes, CAR expression, exhaustion markers, and senescence profiles are illustrated in Figures S4–S6.

### Product release criteria and quality control

Prior to release for clinical use, each CAR T cell product underwent comprehensive in-process and final quality control testing in accordance with predefined release specifications. Product release evaluation included assessment of macroscopic appearance, CD3^+^ T cell purity, cell viability, percentage of CAR expression by flow cytometry, and total viable CAR T cell dose. Functional potency was assessed using an *in vitro* cytotoxicity assay against CD19-positive target cells. Safety testing included sterility assessment by microbial culture, mycoplasma detection, and endotoxin quantification. Evaluation for replication-competent lentivirus and vector copy number per cell was performed using digital droplet PCR to ensure compliance with predefined safety thresholds. The target dose of CD19 CAR T cells was 2 × 10^6^ CAR-positive T cells per kilogram of body weight, with a maximum total dose capped at 200 × 10^6^ CAR-positive T cells. For patient #1, the target dose was 1 × 10^6^ CAR-positive T cells per kilogram, administered prior to the protocol amendment. Products that did not meet predefined specifications were permitted for release and administration at the discretion of the treating physician.

### Lymphodepletion and CAR T cell infusion

Per protocol and institutional practice in Thailand, patients were admitted to a dedicated transplant and cellular therapy inpatient unit equipped with a centralized high efficiency particulate air (HEPA)-filtered positive-pressure laminar airflow system. Lymphodepleting (LD) chemotherapy was administered according to protocol and in parallel with the CAR T cell manufacturing process. LD chemotherapy includes i.v. fludarabine (30 mg/m^2^) and cyclophosphamide (500 mg/m^2^) for 3 days prior to CAR T cell infusion. Subsequently, non-cryopreserved CAR T cell infusion was scheduled 2 days after completion of LD chemotherapy.

### Safety monitoring and efficacy assessment

After CAR T cell infusion, patients remained hospitalized for at least 14 days for monitoring and management of acute toxicities. In the absence of significant adverse events, patients subsequently transitioned to outpatient monitoring and surveillance. CRS and ICANS were diagnosed and managed according to the American Society for Transplantation and Cellular Therapy (ASTCT) consensus criteria.^39^ CAR T cell-associated adverse events were prospectively captured and graded according to the Common Terminology Criteria for Adverse Events (CTCAEs), v.5.0.^40^ Hematologic toxicities, including neutropenia and thrombocytopenia, were classified according to the original neutropenic ICAHT criteria proposed by the EHA and the EBMT consensus criteria,^41^ along with the thrombocytopenic-ICAHT classification.^42^

### Endpoints and assessments

CAR T cell persistence was assessed by multiparametric flow cytometry using a labeled recombinant Fc-tagged CD19 protein conjugated with R-phycoerythrin (R-PE) to detect circulating CAR T cells in peripheral blood samples collected on days 7, 14, 21, and 28, as well as at 3, 6, and 12 months after CAR T cell infusion. Peripheral B cell levels were concurrently quantified by flow cytometry as CD19^+^ lymphocytes to evaluate on-target B cell depletion and recovery following CAR T cell therapy. Details of the flow cytometry gating strategy and antibodies used for CAR T cell and B cell detection are provided in the supplemental information. Inflammatory cytokines and related biomarkers, including interleukin-6 (IL-6), interleukin-1 (IL-1), tumor necrosis factor-α (TNF-α), C-reactive protein (CRP), ferritin, fibrinogen, and D-dimer, were serially assessed at baseline (day 0) and on days 7 and 14.

Response to CAR T cell therapy was evaluated using the positron emission tomography/computed tomography (PET/CT)-based Lugano classification criteria for lymphoma response^43^ and reported as ORR, CMR, partial metabolic response (PMR), and non-response or progressive disease. PET-CT was performed at prespecified time points, including day 30, day 60, day 90, 3 months, 6 months, and 1 year after CAR T cell therapy. EFS and OS were defined according to standard criteria. EFS was defined as the time from the first CAR T cell infusion to the earliest occurrence of documented disease relapse or progression, or death from any cause, whichever occurred first. OS was defined as the time from CAR T cell infusion to death from any cause. Disease response was evaluated in accordance with the Lugano classification criteria. Non-relapse mortality (NRM) was defined as death from causes unrelated to lymphoma, with disease relapse treated as a competing event. Expense for CAR T cell manufacturing, including inpatient care and outpatient follow-up through day +90 or until disease progression (whichever occurred first), was calculated using a conversion rate of 32 Thai Baht per 1 US Dollar (USD).

### Statistical analysis

Categorical variables were summarized as counts and percentages, while continuous variables were reported as medians with ranges or IQRs. Event rates for dichotomous outcomes, including ORR, CRS, and ICANS, were estimated along with exact 95% CIs using Fisher’s exact method. Kaplan-Meier estimates were provided for time-to-event endpoints.

Statistical analyses were performed using Stata v.18 (Stata; StataCorp, College Station, TX, USA) and R software (v.4.5.2; R Foundation for Statistical Computing, Vienna, Austria). Data visualization and figure generation were conducted using R (v.4.5.2) and GraphPad Prism v.8 (GraphPad Software, San Diego, CA, USA).

## Data and code availability

The authors affirm that all data underlying the findings of this study are presented within the article and its supplemental information. Additional data may be obtained from the corresponding author upon request.

## Acknowledgments

We express our sincere gratitude to the patients and their families for their trust and participation in this study. We also thank the CAR T cell manufacturing team and all clinical support personnel for their invaluable contributions to the development and delivery of this therapy. We are especially grateful to the nursing staff for their tireless dedication and compassionate care throughout the patients’ treatment journey. The Center of Excellence in Cellular Immunotherapy, Chulalongkorn University, received infrastructure support and developmental funding from the Office of the National Economic and Social Development Council of Thailand (grant no. N35E690030). We also acknowledge the generous philanthropic contributions that made the compassionate use program possible.

## Author contributions

K.S. proposed the project, participated in CAR T cell manufacturing, and drafted the study protocol; O.S. collected and retrieved clinical data, provided patient care, and drafted the manuscript. S.T. co-proposed the project, oversaw CAR T cell production, and established the manufacturing system and quality control processes; C.C., K.C., M.S., T.A., and U.B. contributed to patient care and approved the manuscript; P.W. supervised the leukapheresis procedures and approved the manuscript; N.H. supervised the project, secured funding support, and approved the manuscript; K.W. co-proposed the project, retrieved and curated clinical data, led the clinical service and patient care, co-drafted and revised the manuscript, and supervised the overall conduct of the project from patient enrollment to manufacturing and clinical management. All authors reviewed and approved the final manuscript.

## Declaration of interests

All authors declare that they have no relevant conflicts of interest related to this study.

## Declaration of generative AI and AI-assisted technologies in the writing process

The authors used ChatGPT solely to improve the grammar and clarity of the manuscript. All other contents are original and was not generated by artificial intelligence. The authors reviewed all the content and take full responsibility for the content of the published article.

## Supplemental information

Document S1. Figures S1–S6 and Tables S1–S4

Document S2. Article plus supplemental information
