# Supplementary material for: Feasibility and outcomes of decentralized point-of-care CD19 CAR T cell for relapsed large B cell lymphoma in resource-limited settings
Source: Mol Ther Oncol. 2026 Jun 17;34(3):201278. doi: 10.1016/j.omton.2026.201278 (PMC13333335; doi:10.1016/j.omton.2026.201278)
Supplement: Document S1. Figures S1–S6 and Tables S1–S4 [file mmc1.pdf]

OMTON, Volume *34*

Supplemental information

Feasibility and outcomes of decentralized point-of-care CD19 CAR T cell for relapsed large B cell lymphoma in resource-limited settings

Koramit Suppipat, Supannikar Tawinwung, Ornnicha Sathitakorn, Chantiya Chanswangphuwana, Kanhatai Chiengthong, Mutita Surakijboworn, Thiti Asawapanumas, Phandee Watanaboonyongcharoen, Palada Pitakkitnukun, Manaschanok Tippawan, Supanat Kumjan, Udomsak Bunworasate, Nattiya Hirankarn, and Kitsada Wudhikarn

SUPPLEMENTAL TABLES

Table S1: The detailed characteristics of the final CAR T-cell products for each individual patient in the cohort.

Parameter	Apperance	CD3+ (%)	Viability (%)	CAR (%)	Total CAR ($x10^6$ cells) [$x10^6$ cells/kg]	Killing (%)	Sterility	Mycoplasma	Endotoxin (EU/ml)	RCL	VCN (copies/cells)
**Method**	Visual inspection	Flow cytometry	AO-PI staining	Flow cytometry	Calculation	Flow cytometry	BD Bactec	Q-PCR	Endosafe PTS	DD-PCR	DD-PCR
**Specification**	White to yellowish, slightly hazy to cloudy cell suspension, without visible particles	≥ 80	≥ 70	≥ 2	≥ 0.5	≥ 20	Day 5, Day 8, and Day 12 of culture: No growth of Aerobe, Anaerobe and Fungus	Negative	≤ 5.00	ND	< 5
**P01**	Conform	93	87	21	55 [1.1]	90	No growth	Negative	≤ 5.00	ND	1.52
**P02**	Conform	97	97	38	338 [6.9]	98	No growth	Negative	≤ 5.00	ND	1.74
**P03**	Conform	81	93	22	280 [1.9]	40	No growth	Negative	≤ 5.00	ND	1.13
**P04**	Conform	99	95	46	496 [8.4]	92	No growth	Negative	≤ 5.00	ND	1.28
**P05**	Conform	99	97	15	651 [8.9]	80	No growth	Negative	≤ 5.00	ND	1.34
**P06**	Conform	99	99	45	56 [0.7]	50	No growth	Negative	≤ 5.00	ND	2.24
**P07**	Conform	99	95	19	177 [3.5]	25	No growth	Negative	≤ 5.00	ND	1.32
**P08**	Conform	99	91	36	908 [18.9]	82	No growth	Negative	≤ 5.00	ND	1.66
**P09**	Conform	99	96	40	2459 [41]	99	No growth	Negative	≤ 5.00	ND	1.95
**P10**	Conform	99	94	65	3896 [49.3]	89	No growth	Negative	≤ 5.00	ND	2.45
**P11**	Conform	99	94	30	567 [8.3]	99	No growth	Negative	≤ 5.00	ND	1.71
**P12**	Conform	97	95	36	1873 [27.6]	97	No growth	Negative	≤ 5.00	ND	1.80
**Summary**	N/A	97, 5 (Mean, SD)	95.6,2.2 (Mean, SD)	36, 15-65 (Median, min-max)	531, 55-3896 [8.3, 0.7-49.3] (Median, min-max)	78.4, 25.5 (Mean, SD)	N/A	N/A	N/A	N/A	1.68, 0.40 (Mean, SD)

Abbreviations: CAR – Chimeric Antigen Receptor, RCL - Replication-Competent Lentivirus, VCN - Vector Copy Number, AO-PI - Acridine Orange/Propidium Iodide, PCR - Polymerase chain reaction, PTS - Portable Testing System, DD – Digital Droplet, ND – Negative Droplet, N/A – Not applicable, SD – Standard Deviation

Table S2: Summary of Toxicities in 12 patients treated with Prodigy CliniMACS CD19 CAR T-Cell

**Parameters**	**N = 12 (%)**
**Cytokine release syndrome (CRS)**	
- Any grade	5 (41.67%)
- Grade $\geq$ 3	1 (8.33%)
- Median time to CRS onset (range, days)	1 (1-5)
**Immune effector cell associate neurotoxicity syndrome (ICANS)**	
- Any grade	1 (8.33%)
- Grade $\geq$ 3	0 (0%)
**Treatment for CRS or ICANs**	
- Tocilizumab without corticosteroid	2 (16.67%)
- Corticosteroid without tocilizumab	0 (0%)
**Cytopenia before day 30**	
- ANC < 1000 cells/μL	9 (75%)
- Platelet count < 75 $x10^3$/μL	6 (50%)
- Hb < 8 g/dL	0 (0%)
**Prolonged cytopenia (≥30 days after CAR T-cell infusion)**	
- Neutropenia	0 (0%)
- Thrombocytopenia	1 (8.33%)
**Clinically significant infection requiring treatment**	
- Bacterial infection	0 (0%)
- Viral infection	1 (8.33%)
- Fungal infection	0 (0%)

Abbreviations: CAR – Chimeric Antigen Receptor, ANC – Absolute Neutrophil Count, Hb - Hemoglobin

Table S3: List of proteins and antibodies used in this study.

Marker	Conjugate	Clone, specificity	Information (Species, chain)	Company	Catalog#	Volume in µl/sample
**Surface antibodies**						
**Subset**						
CD3	PerCP-Cy™5.5	UCHT1	Mouse BALB/c IgG1, κ	BD Biosciences	560835	1
CD4	BB515	RPA-T4	Mouse IgG1, κ	BD Biosciences	564419	1
CD8	PE	SK1	Mouse BALB/c IgG1, κ	BD Biosciences	340046	1
CD14	APC-H7	MφP9	Mouse BALB/c IgG2b, κ	BD Biosciences	560180	1
CD19	APC	HIB19	Mouse IgG1, κ	BD Biosciences	555415	10
CD56	BV421	NCAM16.2	Mouse BALB/c IgG2b, κ	BD Biosciences	562751	1
**Efficiency**						
Anti-human CD19 CAR biotin	-	-	Recombinant human IgG1	Miltenyi Biotec	130-129-550	1
Biotin Antibody	PE	REA746	Recombinant human IgG1	Miltenyi Biotec	130-110-951	1
CD3	APC	OKT3	Mouse IgG2a, κ	BioLegend	317318	1
**Phenotype/exhaustion/senescence**						
Mouse IgG2b, κ	FITC	MPC-11	Mouse IgG2b, κ	BioLegend	400310	1
Mouse IgG2a, κ	PE	MOPC-173	Mouse IgG2a, κ	BioLegend	400214	1
Mouse IgG1 κ	PE-Cy™7	MOPC-21	Mouse IgG1, κ	BD Biosciences	565573	1
Mouse IgG2b, κ	BV421	27-35	Mouse C.SW IgG2b, κ	BD Biosciences	562748	1
Mouse IgM, κ	BB515	G155-228	Mouse BALB/c IgM, κ	BD Biosciences	564680	1
CD197 (CCR7)	PE	G043H7	Mouse IgG2a, κ	BioLegend	353204	1
CD223 (LAG-3)	BV421	T47-530	Mouse IgG1, κ	BD Biosciences	565720	1
CD27 (TNFRSF7)	PE-Cy™7	O323	Mouse IgG1, κ	BD Biosciences	567289	1
CD279 (PD-1)	PE-Cy™7	EH12.1	Mouse IgG1, κ	BD Biosciences	561272	1
CD28	BV421	CD28.2	Mouse C3H x BALB/c IgG1, κ	BD Biosciences	562613	1
CD336 (TIM-3)	BB515	7D3	Mouse IgG1, κ	BD Biosciences	565568	1
CD45RA	FITC	HI100	Mouse IgG2b, κ	BioLegend	304106	1
CD57	BB515	NK-1	Mouse IgM, κ	BD Biosciences	565285	1
CD62L	BV421	DREG-56	Mouse IgG1, κ	BD Biosciences	563862	1
Mouse IgG2a, κ	Brilliant Violet 510™	MOPC-173	Mouse IgG2a, κ	BioLegend	400268	1
TIGIT (VSTM3)	Brilliant Violet 510™	A15153G	Mouse IgG2a, κ	BioLegend	372738	1
**Cytotoxicity**						
CD3	PE	OKT3	Mouse IgG2a, κ	BD Biosciences	317308	1
**Cell dyes**						
BD Pharmingen™ 7-AAD				BD Biosciences	559925	2

Table S4: List of publications of Miltenyi Biotec CD19 LVV Derived CAR T-Cell in Hematologic Malignancy. Updated May 2026. Publications:

Year	Article	Reference
2026	1. Liem Thanh Nguyen, Duy D. Nguyen, Quoc Khanh Bach, Lan T.M. Dao, Trang Thi Kieu Phan, Hoang - Phuong Nguyen, Hong-Nhung Dao, Trang H. Pham, Phuong T. Pham, Hien T. Mai, Viet Huong T. Pham, Thanh Mai T. Nguyen, Van Binh Le, Nam Lam Phung, Ngoc Quang Nguyen, Michelle L. Hermiston, Quynh Lan Phan, Do Quang Trung Nguyen, Lan Mai, Quoc Nhat Nguyen, Van T. Hoang. Outcomes of point-of-care manufactured CAR T-cell therapy for B-cell acute lymphoblastic leukemia and non-Hodgkin lymphoma in Vietnam. Molecular Therapy Oncology 34 (1), 201156. (2026). https://doi.org/10.1016/j.omton.2026.201156	21
2025	1. Francesca Del Bufalo, Marco Becilli, Chiara Rosignoli, Pietro Merli, Mattia Algeri, Daria Pagliara, Federica Galaverna, Michele Massa, Valeria Paganelli, Maria Giuseppina Cefalo, Linda Hanssens, Valentina Bertaina, Giuseppina Li Pira, Giovanna Leone, Matilde Sinibaldi, Stefano Di Cecca, Laura Iaffaldano, Valentina Fustaino, Biagio De Angelis, Concetta Quintarelli, Franco Locatelli; Point-of-care fresh CAR T cells for pediatric or young adult BCP-ALL that is relapsed/refractory or in very-high-risk first relapse. Blood Adv. 2025 Oct 14;9(19):5091-5102. DOI: 10.1182/bloodadvances.2025016181	22
	2. Armin Ghobadi, Paolo F. Caimi, Jane S. Reese, Krishna Goparaju, Martina di Trani, Julie Ritchey, Zachary Jackson, Benjamin Tomlinson, Jennifer M. Schiavone, Sarah Kleinsorge-Block, Kayla Zamborsky, Linda Eissenberg, Dina Schneider, Kirsten M. Boughan, Emily C. Zabor, Leland Metheny, Molly Gallogly, Winfried Kruger, Michael Kadan, Andrew Worden A.S, Ashish Sharma, Brenda W. Cooper, Folashade Otegbeye, Rafick P. Sekaly, David N. Wald, Carmelo Carlo-Stella, John DiPersio, Rimas Orentas, Boro Dropulic, and Marcos de Lima. Treatment of non-Hodgkin lymphoma with point-of-care manufactured CAR T cells: a dual institution, phase 1 trial. eClinicalMedicine, 2025 Mar, Volume 81, 103138 DOI: https://doi.org/10.1016/j.eclinm.2025.103138	23

	3. Rosario Salazar-Riojas, Dalila M. Alvarado-Navarro, Yair O. Chávez-Estrada, Ana K. Hernández-Navarro, Martha B. Ake-Uc, Nidia K. Moncada-Saucedo, José C. Jaime-Pérez, SofíaI. Quezada-Ramírez, Anna C. Rodriguez-Zuñiga, David Gómez-Almaguer, Andrés Gómez-De León. Decentralized Point-of-Care Manufacturing of CD19 Chimeric Antigen Receptor T Cells in Mexico. JCO Glob Oncol 11, e2400581(2025) https://DOI:10.1200/GO-24-00581<br><br>4. Hamenth Kumar Palani, Arun Kumar Arunachalam, Uday Kulkarni, Mohammed Yasar, Arvind Venkatraman, Swathy Palanikumar, Reeshma Nair Radhakrishnan, Majeela Solomon, Abirami Rajasekaran, Aniket Bankar, Phaneendra Venkateswara Rao Datari, Sushil Selvaraja, Anu Korula, Pradyot Dash, Dina Schneider, Louisa Wirthlin, Aby Abraham, Biju George, Vikram Mathews. Safety, efficacy and total cost of point-of-care manufactured anti-CD19 CAR-T cell therapy in India: VELCART trial. Molecular Therapy Oncology, Volume 33, Issue 2, 200977 DOI: https://10.1016/j.omton.2025.200977<br><br>5. Malakhova E, Pershin D, Kulakovskaya E, Vedmedskaia V, Fadeeva M, Lodoeva O, Sozonova T, Muzalevskii Y, Kazachenok A, Belchikov V, Shelikhova L, Molostova O, Volkov D, Maschan M. Extended characterization of anti-CD19 CAR T cell products manufactured at the point of care using the CliniMACS Prodigy system: comparison of donor sources and process duration. Cytotherapy 2024 Jun;26(6):567-578. doi: 10.1016/j.jcyt.2024.02.025. PMID: 38493403.<br><br>6. Luanpitpong S, Klaihmon P, Janan M, Kungwankiattichai S, Owattanapanich W, Kunacheewa C, Chanthateyanonth S, Donsakul N, U-Pratya Y, Warindpong T, Kittivorapart J, Permpikul P, Issaragrisil S. Point-of-care manufacturing of anti-CD19 CAR-T cells using a closed production platform: Experiences of an academic in Thailand. Mol Ther Oncol. 2024 Oct 5;32(4):200889. doi: 10.1016/j.omton.2024.200889. PMID: 39507317; PMCID: PMC11539415.	24<br><br>25<br><br>26<br><br>27
2022	1. Hamenth Kumar Palani, Arun Kumar Arunachalam, Mohammed Yasar, Arvind Venkatraman, Uday Kulkarni, Sharon Anbumalar Lionel, Sushil Selvarajan, Anu Korula, Aby Abraham, Biju George, Jennifer E. Adair, Rimas Orentas, Boro Dropulic and Vikram Mathews. Decentralized manufacturing of anti CD19 CAR-T cells using CliniMACS Prodigy®: real-world experience and cost analysis in India. Bone Marrow Transplantation 58, 160-167 (2023). https://doi.org/10.1038/s41409-022-01866-5	28

2021	1. Caimi PF, Pacheco Sanchez G, Sharma A, Otegbeye F, Ahmed N, Rojas P, Patel S, Kleinsorge Block S, Schiavone J, Zamborsky K, Boughan K, Hillian A, Reese-Koc J, Maschan M, Dropulic B, Sekaly R-P and de Lima M (2021) Prophylactic Tocilizumab Prior to Anti-CD19 CAR-T Cell Therapy for Non-Hodgkin Lymphoma. Front. Immunol. 12:745320.<br>2. Maschan, M., Caimi, P.F., Reese-Koc, J. et al. Multiple site place-of-care manufactured anti-CD19 CAR-T cells induce high remission rates in B-cell malignancy patients. Nat Commun 12, 7200 (2021).	29<br>30
2020	1. Jackson Z, Roe A, Sharma AA, Lopes FBTP, Talla A, Kleinsorge-Block S, Zamborsky K, Schiavone J, Manjappa S, Schauner R, Lee G, Liu R, Caimi PF, Xiong Y, Krueger W, Worden A, Kadan M, Schneider D, Orentas R, Dropulic B, Sekaly R-P, de Lima M, Wald DN and Reese JS (2020). Automated Manufacture of Autologous CD19 CAR-T Cells for Treatment of Non-hodgkin Lymphoma. Front. Immunol. 11:1941.	19
2018	1. Fenlu Zhu, Nirav Shah, Huiqing Xu, Dina Schneider, Rimas Orentas, Boro Dropulic, Parameswaran Hari, Carolyn A. Keever-Taylor. Closed-system manufacturing of CD19 and dual-targeted CD20/19 chimeric antigen receptor T cells using the CliniMACS Prodigy device at an academic medical center, Cytotherapy VOLUME 20, ISSUE 3, P394-406, MARCH 01, 2018<br>2. Wei Zhang, Kimberly R Jordan, Brian Schulte, Enkhtsetseg Purev. Characterization of clinical grade CD19 chimeric antigen receptor T cells produced using automated CliniMACS Prodigy system. Drug Design, Development and Therapy 2018:12 3343–3356.	31<br>32

Websites

Through a partnership with Lentigen (now Miltenyi), Marco and his team developed a twist on a familiar CAR construct. The slide below shows the construct with a 4-1BB co-stimulator molecule, the difference is the transmembrane domain that uses a TNF receptor superfamily. The team went from manufacturing ideas to treating their first patient in under a year, who had high-risk lymphomas and had already received more than two lines of therapy. The process involved an IL-7/IL-15 culture and was done in Miltenyi's Prodigy platform.

From Expensive Treatments to Point-of-Care: Hospital-Made CAR-T Cell Therapy Offers Hope for Wider Access and Innovation (title21.com)

SUPPLEMENTAL FIGURES

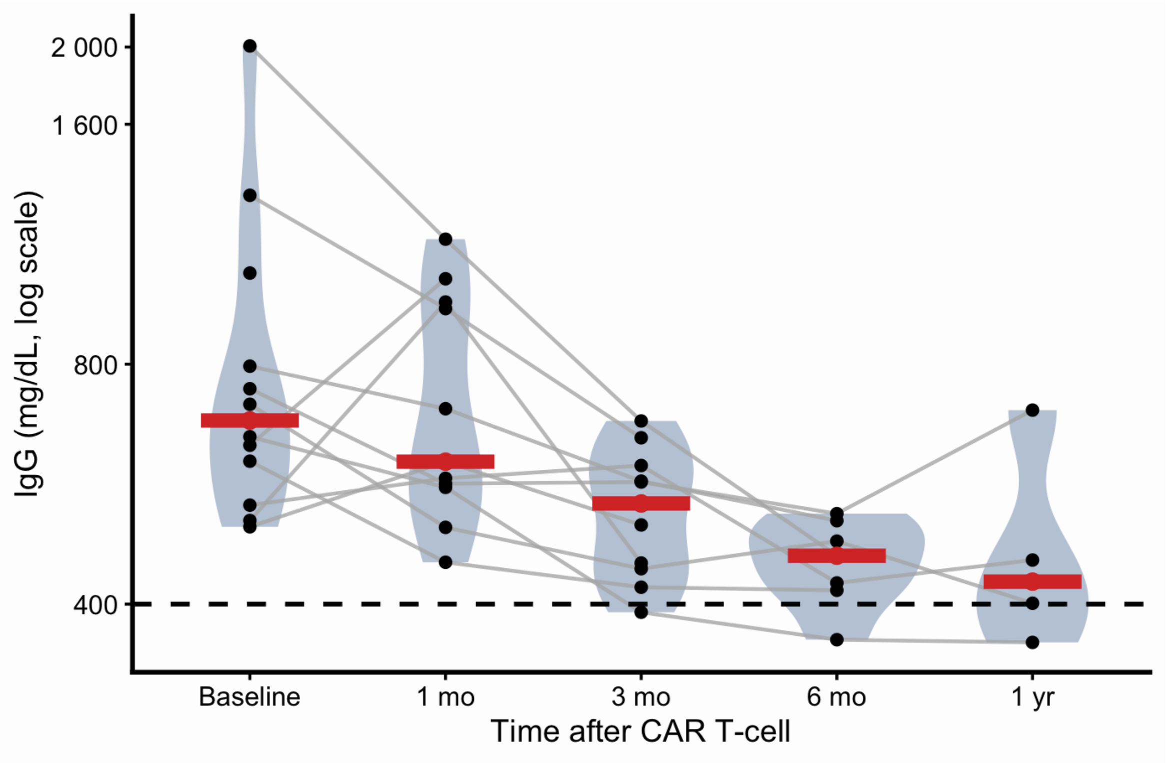

Figure S1: Longitudinal trends in serum IgG concentrations across serial timepoints

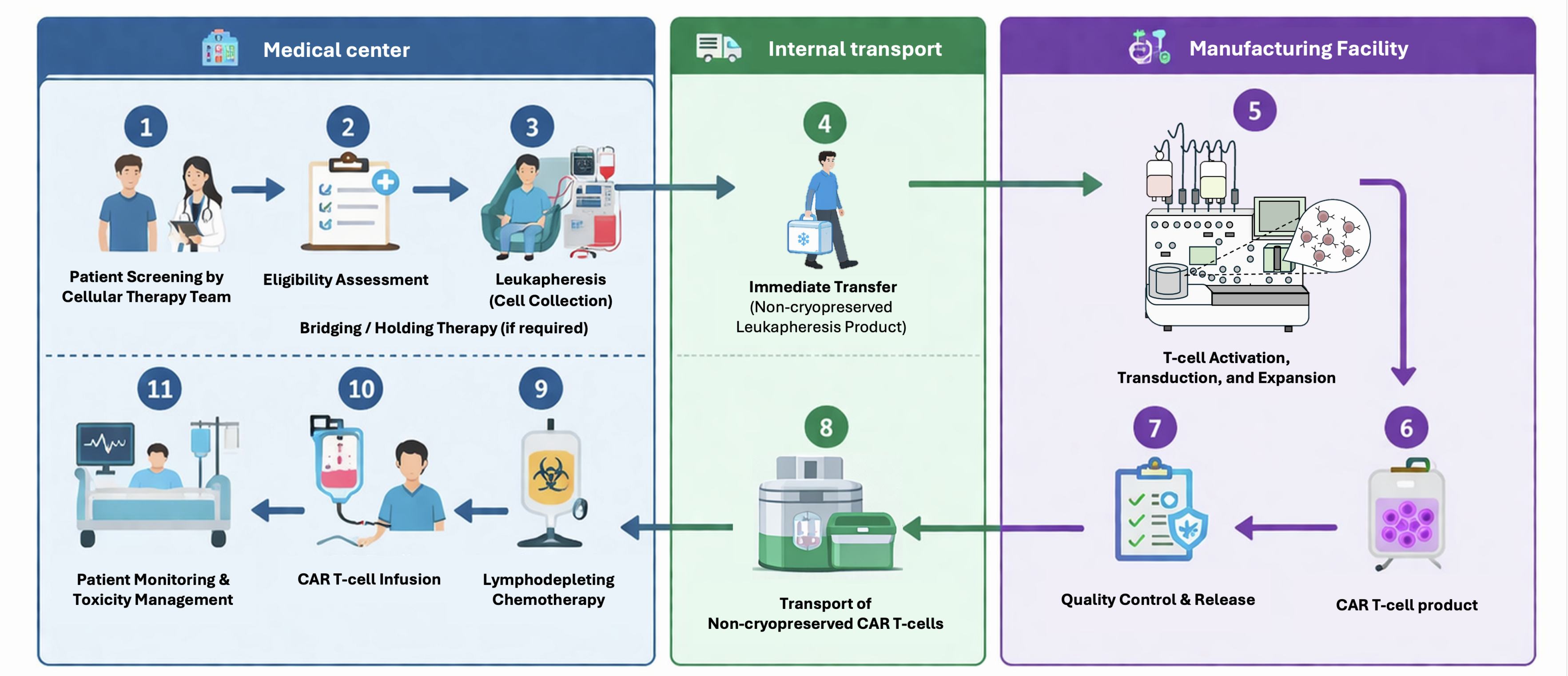

Figure S2: Schematic overview of the stepwise logistical workflow for CAR T-cell service and decentralized manufacturing at King Chulalongkorn Memorial Hospital

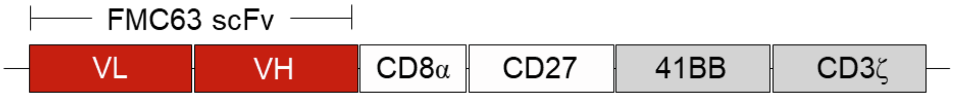

Figure S3: CAR construct design - Schematic representation of the linear CAR vector construct

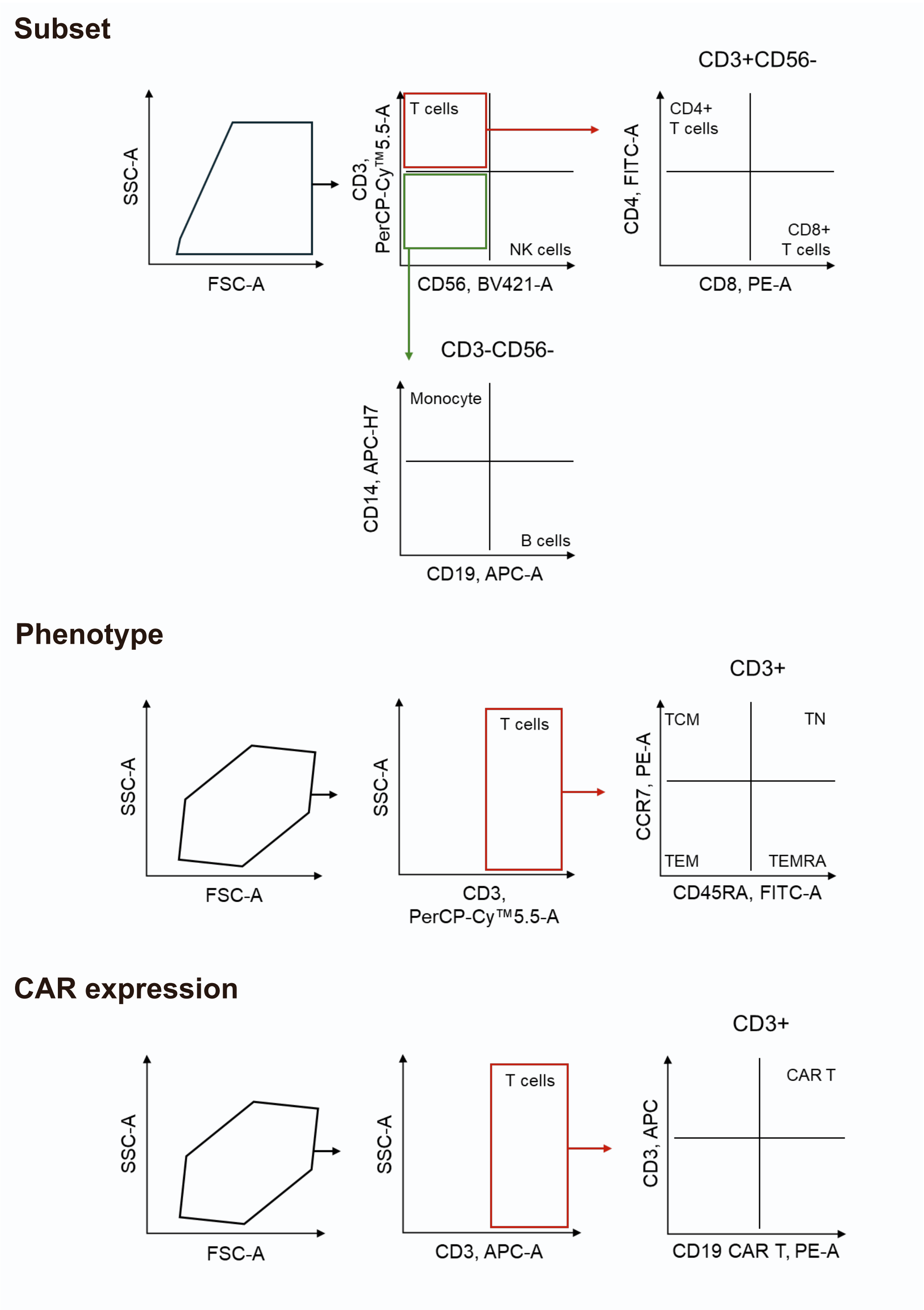

Figure S4: Flow Cytometric Gating Strategies for the Characterization of T-Cell Subsets, Phenotypes, and CAR Expression

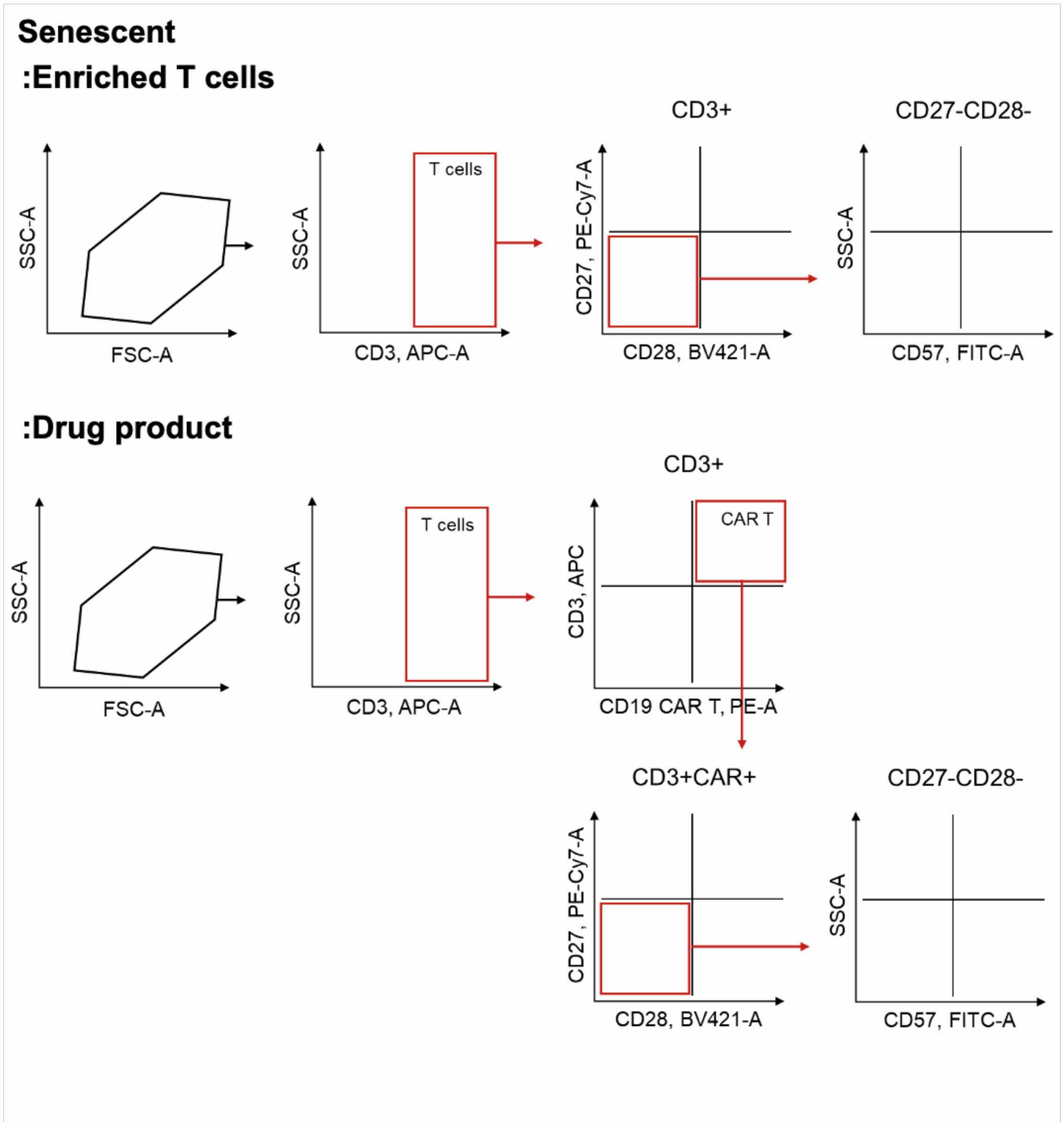

Figure S5: Flow Cytometry Gating Strategy for Senescence Profiling in Enriched T Cells and Final CAR T-Cell Product

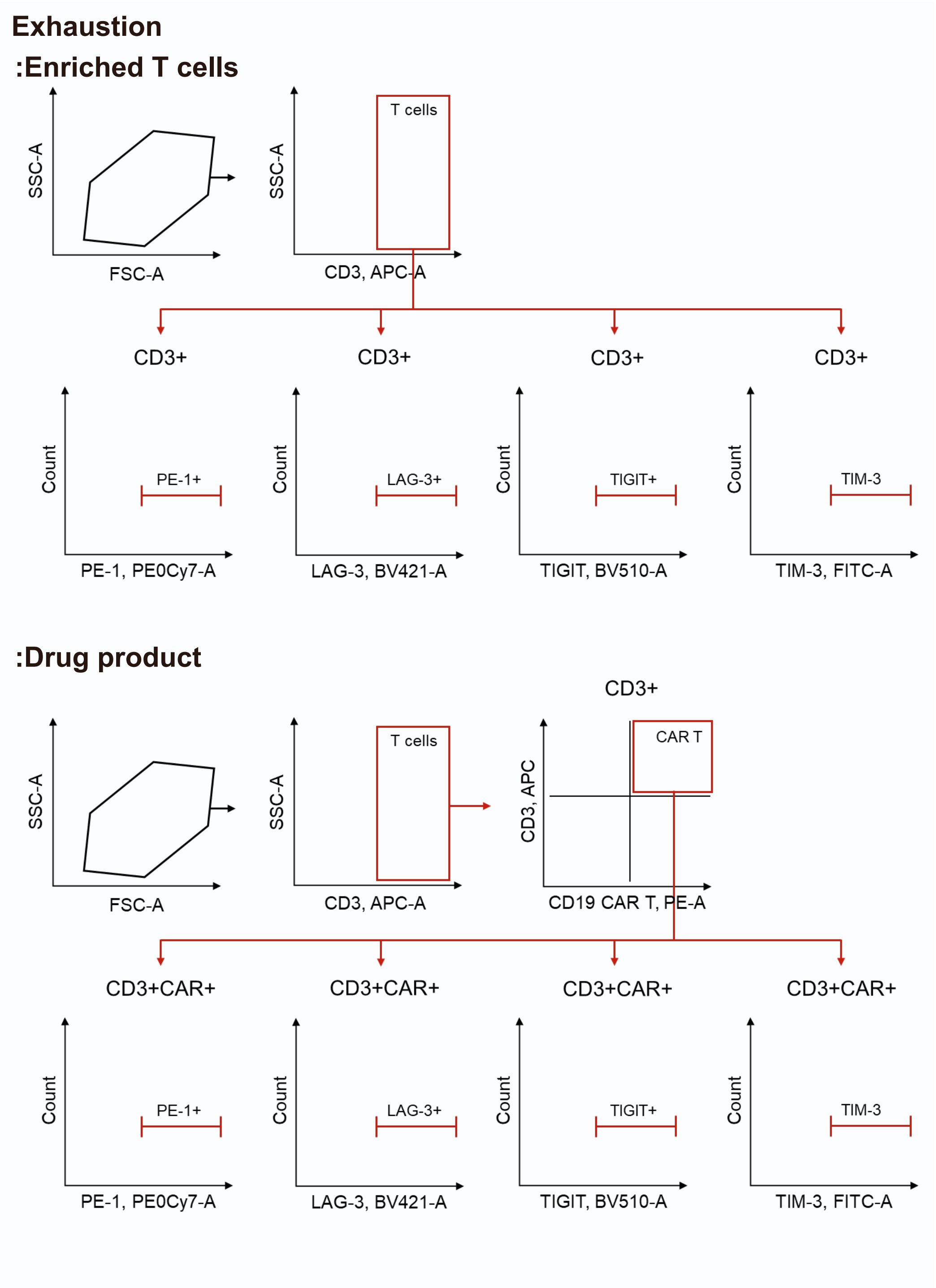

Figure S6: Flow Cytometry Gating Strategy for Exhaustion Marker Analysis in Enriched T Cells and Final CAR T-Cell Product
